# MORC2 promotes cell growth and metastasis in human cholangiocarcinoma and is negatively regulated by miR-186-5p

**DOI:** 10.18632/aging.102003

**Published:** 2019-06-09

**Authors:** Guanqun Liao, Xiaopeng Liu, Dehai Wu, Fangting Duan, Xueyi Xie, Shunqian Wen, Yiqun Li, Shengping Li

**Affiliations:** 1 State Key Laboratory of Oncology in South China, Collaborative Innovation Center for Cancer Medicine, Sun Yat-sen University Cancer Center, Guangzhou 510060, P. R. China; 2 Department of Hepatobiliary and Pancreatic Oncology, Sun Yat-sen University Cancer Center, Guangzhou 510060, P. R. China; 3 Department of Hepatobiliary Surgery, Foshan Hospital Affiliated to Southern Medical University, Foshan 528000, P. R. China; 4 Department of Hepatopancreatobiliary Surgery, First Affiliated Hospital of Harbin Medical University, Harbin 528001, P. R. China

**Keywords:** MORC2, cholangiocarcinoma, proliferation, metastasis, miR-186-5p

## Abstract

Microrchidia family CW-type zinc finger 2 (MORC2) is a ubiquitously expressed protein that contributes to chromatin remodeling, DNA repair, and lipogenesis. However, its role in cholangiocarcinoma (CCA) remains largely unknown. The aim of this study was to investigate the expression profile of MORC2 and its potential functions in CCA progression. The results showed that MORC2 was upregulated in human CCA specimens and cell lines. MORC2 expression was significantly associated with serum CA19-9 levels (P = 0.009), TNM stage (P = 0.003) and lymph node invasion (P = 0.004). Furthermore, high MORC2 expression was associated with poor 5-year survival (P = 0.016). Functional experiments revealed that MORC2 knockdown could suppress CCA cell proliferation, migration, and invasion both *in vivo* and *in vitro*. Mechanically, we found that MORC2 promoted CCA cell metastasis through the EMT process and enhanced proliferation via the Akt signaling pathway. Moreover, MORC2 was negatively regulated by miR-186-5p. MiR-186-5p could influence CCA cell proliferation, migration and metastasis by regulating MORC2. Taken together, the findings of this study demonstrated the oncogenic role of MORC2 in CCA tumorigenesis and metastasis, and clarified an underlying regulatory mechanism mediating MORC2 upregulation, which may provide a novel therapeutic target in CCA treatment.

## INTRODUCTION

Cholangiocarcinoma (CCA) is a highly aggressive cancer derived from bile duct epithelial cells. Several reports have shown that the incidence and mortality of CCA is increasing worldwide [1]. Surgical resection remains the most effective treatment for CCA patients. However, most patients present with advanced disease, providing few chances for surgery. New adjuvant therapies are urgently required. However, the poor understanding of the molecular mechanism underlying CCA tumorigenesis impedes the development of new agents. Hence, identifying novel targets is extremely essential to improving the survival of patients with CCA.

Microrchidia family CW-type zinc finger 2 (MORC2) is a highly conserved nuclear matrix protein, that is characterized by the presence of a zinc finger type CW domain with a nuclear localization signal, an ATPase domain, and several coiled-coil domains [2, 3]. MORC2 plays important roles in diverse biological processes such as chromatin remodeling, DNA damage repair, and lipogenesis [4, 5]. Recent work has demonstrated that MORC2 may act as an oncogene in several cancer types. It has been documented that MORC2 facilitates the recruitment of EZH2 and suppresses the transcriptional activity of ArgBP2 [6]. Mutant MORC2 is also involved in the metastatic progression of triple-negative breast cancer (TNBC) by regulating CD44 splicing [7]. Additionally, MORC2 is essential for maintaining cancer stemness, sorafenib resistance and tumorigenesis of hepatocellular carcinoma (HCC) by repressing two critical components involved in the Hippo signaling pathway, NF2 and KIBRA, at the transcriptional level [8]. Moreover, MORC2 promotes colorectal cancer (CRC) cell migration and invasion by inhibiting the promoter activity of NDRG1 [9]. However, to date, the potential function of MORC2 in CCA remains largely unknown.

In the current study, we examined the expression profile, functional role and underlying molecular mechanism of MORC2 in CCA. We first reported that MORC2 was significantly upregulated in CCA specimens. Knockdown of MORC attenuated tumor cell proliferation, migration and invasion, indicating that MORC2 might act as an oncogene involved in the development of human CCA. Furthermore, we found that microRNA (miR)-186-5p, a well-characterized tumor suppressor [10, 11], can directly bind to the 3′-UTR of MORC2 mRNA, and may function as a scaffold for MORC2, thereby repressing the expression of MORC2 at the post-transcriptional level.

## RESULTS

### MORC2 is upregulated in CCA and is associated with worse outcome

**Figure 1:**
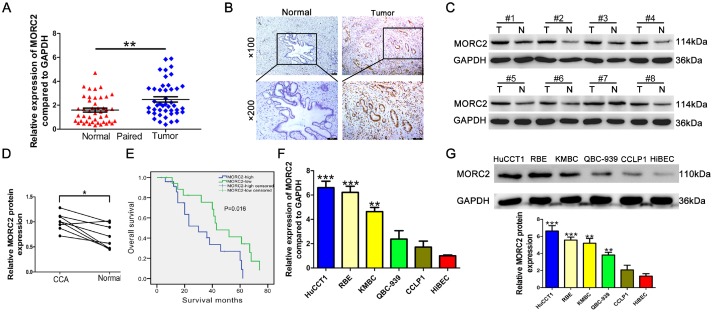
**MORC2 is highly expressed in CCA and is associated with poor prognosis**. (**A**) The expression of MORC2 mRNA was determined in 44 pairs of CCA samples. (**B**) The expression of MORC2 in paired CCA samples from cohort 2 was confirmed by immunohistochemical staining. Representative examples of MORC2 staining are shown. (**C**) The protein expression of MORC2 was examined in 8 pairs of CCA tumor tissues (T) and corresponding normal tissues (N). (**D**) The relevant density of MORC2 protein was compared, and the results are shown in the scatter plot. (**E**) Kaplan-Meier survival analyses were conducted to evaluate the influence of MORC2 on overall survival. (**F**–**G**) mRNA and protein expression levels of MORC2 were evaluated by RT-qPCR and western blotting in six CCA cell lines. ***P < 0.001, **P < 0.01, *P < 0.05.

We first performed qRT-PCR to examine the mRNA levels of MORC2 in 44 pairs of CCA specimens (tumor tissue and corresponding nontumorous tissue). The data showed that MORC2 mRNA was highly expressed in CCA specimens compared with that in the corresponding nontumorous tissues (Figure 1A). In addition, the protein levels of MORC2 were examined in CCA specimens and adjacent nontumorous specimens of 44 patients with CCA by immunohistochemistry (IHC). The data revealed that the staining-positive rate in CCA specimens was 65.9% (29/44), which was markedly higher than that13.6% (6/44) in the corresponding adjacent nontumorous tissues (χ2 tests P < 0.024) (Figure 1B). As shown in Table 1, MORC2 expression was significantly associated with serum CA19-9 level (P = 0.009), TNM stage (P = 0.003) and lymph node invasion (P = 0.004). Eight pairs of CCA tissues were used to detect the protein levels of MORC2 by western blot. Consistently, tumor tissues exhibited remarkably higher levels of MORC2 protein than those in noncancerous CCA tissues (Figure 1C–1D). Furthermore, Kaplan-Meier analysis revealed that patients with MORC2-positive tumors have significantly poorer prognosis than their MORC2-negative counterparts (P = 0.003, Figure 1E). Next, we detected MORC2 mRNA expression in five CCA cell lines and HIBEC cells. The results revealed that the mRNA levels of MORC2 were markedly upregulated in CCA cells compared to those in the HIBECs (Figure 1F). Besides, the expression of MORC2 protein was expressed at a significantly higher level in the CCA cells compared with that in the HIBECs (Figure 1G).

**Table 1:** Association between the expression of MORC2 and clinicopathologic characteristics in 44 patients with CCA.

**Clinical characteristics**	**Total**	**MORC2 expression**		**P-value**
		**Negative-Low**	**High**	
Age				0.908
≤ 60 years	24	8	16	
> 60 years	20	7	13	
Gender				0.939
Male	29	10	19	
Female	15	5	10	
Serum CA19-9 level				0.009
>37 U/ml	29	6	23	
≤37 U/ml	15	9	6	
Histologic differentiation				0.914
Well	13	4	9	
Moderate	18	6	12	
Poor	13	5	8	
TNM stage				0.003
I-II	16	10	6	
III-IV	28	5	23	
Lymph node invasion				0.004
Present	25	4	21	
Absent	19	11	8	

### Knockdown of MORC2 suppresses CCA cell proliferation *in vitro*

**Figure 2:**
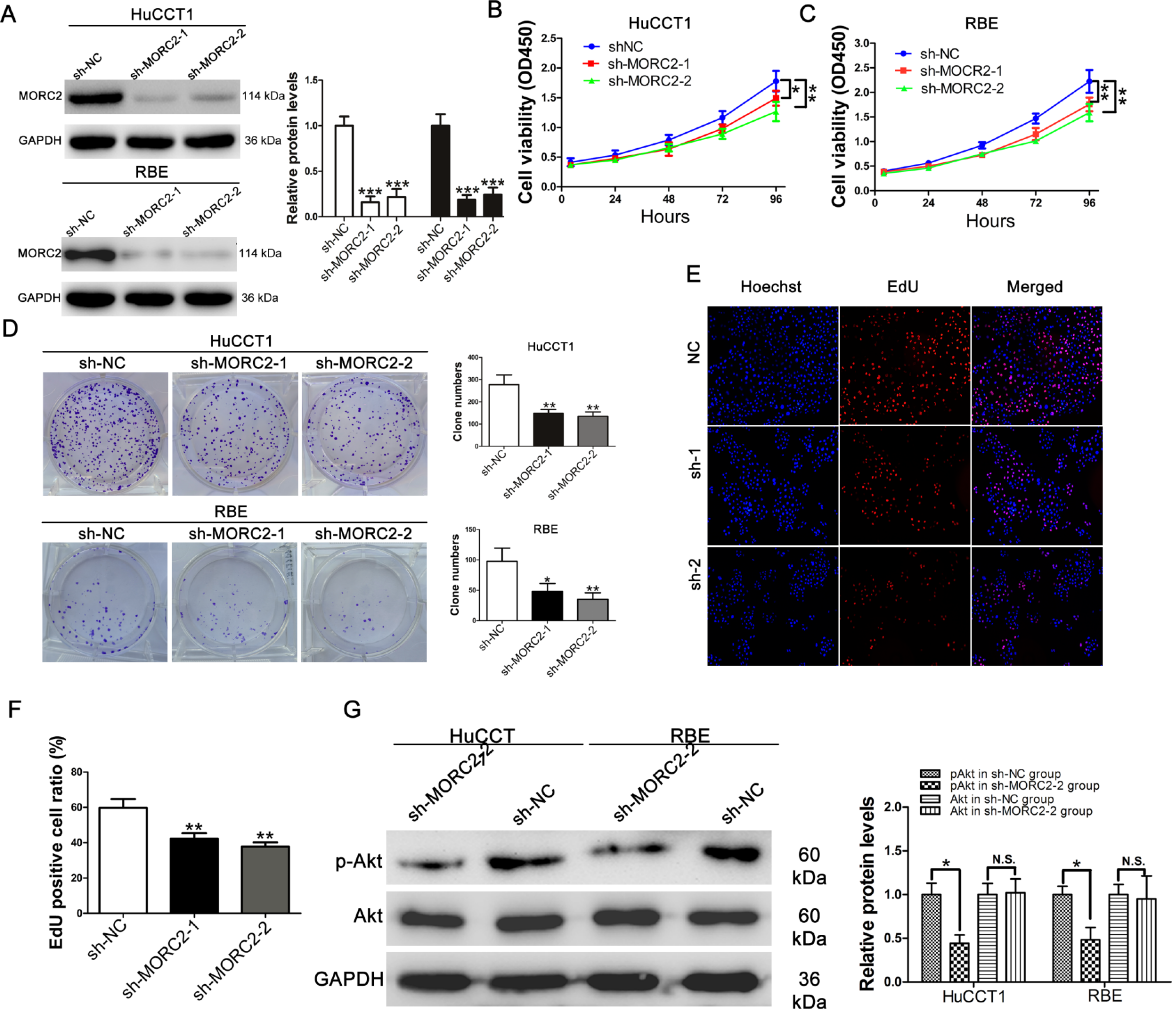
**Knockdown of MORC2 suppresses CCA cells proliferation *in vitro***. (**A**) MORC2 protein levels in stable HuCCT1 and RBE cells. Each cell line was divided into the following three groups: the sh-NC group (cells infected with Lv-sh-CTRL), the sh-MORC2-1 group (cells infected with Lv-sh-MORC2-1) and the sh-MORC2-2 group (cells infected with Lv-sh-MORC2-2). (**B**–**C**) Cell Counting Kit-8 assay of cell proliferation in MORC2 stable knockdown HuCCT1 and RBE cells; cell viability was determined at 24, 48, 72 and 96 h. (**D**) Representative images and quantitative clonogenic analysis of plate colony formation in CCA cells. (**E**–**F**) DNA synthesis was analyzed in CCA cells transfected with the corresponding shRNA via an EdU incorporation assay. (**G**) The expression levels of p-AKT and AKT in MORC2-knockdown HuCCT1 and RBE cells were examined by western blotting. All experiments were performed in triplicate, **P < 0.01, *P < 0.05.

To test the oncogenic activity of MORC2, two cell lines (HuCCT1 and RBE) with higher MORC2 expression were selected to establish stable cell lines with knockdown of MORC2. Compared with the negative control shRNA, the expression of MORC2 protein was significantly decreased by sh-MORC2-1 and sh-MORC2-2 (Figure 2A). Cell counting kit-8 (CCK-8) and plate clone formation assays were conducted to test the effect of MORC2 on the tumor growth of CCA cells. The results revealed that silencing MORC2 in HuCCT1 and RBE cells notably reduced cell viability and clonogenicity (Figure 2B–2D). In addition, an EdU incorporation assay was performed to confirm the above results. As shown in Figure 2E–2F, knockdown of MORC2 attenuated the ratio of EdU-positive HuCCT1 cells. Akt activation is essential for cancer cell proliferation [12–13]. To examine whether MORC2 activated Akt in CCA, thus promoting cell proliferation, western blot analysis was conducted. The data showed that knockdown of MORC2 in HuCCT1 and RBE cells resulted in dramatically decreased phosphorylation of Akt (Figure 2G).

### MORC2 knockdown impairs the migratory and invasive capacities of CCA cells

**Figure 3:**
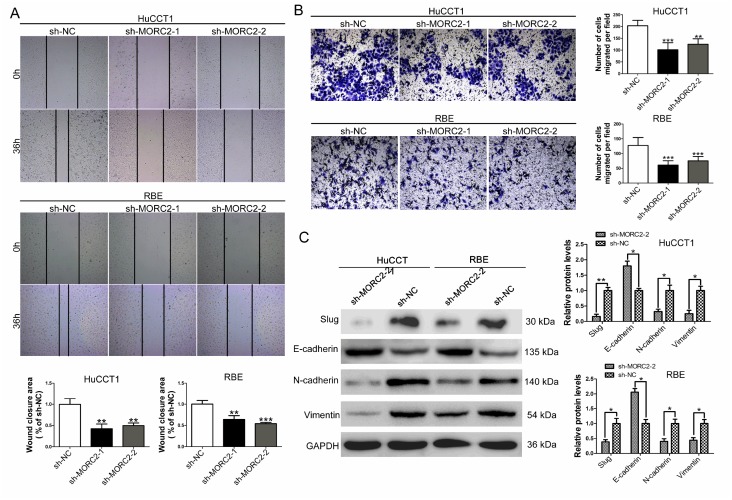
**MORC2 knockdown impairs the migratory and invasive capacities of CCA cells**. (**A**) The wound healing assay was used to determine the migration of MORC2 stable knockdown HuCCT1 and RBE cells. (**B**) Cell invasion was investigated by transwell assays in MORC2 stable knockdown HuCCT1 and RBE cells. (**C**) Slug, E-cadherin, N-cadherin and vimentin protein levels were detected and quantified in MORC2 stable knockdown HuCCT1 and RBE cells by western blotting. All experiments were performed in triplicate, ***P < 0.001, **P < 0.01.

To examine the effect of MORC2 on the capability of migration in HuCCT1 and RBE cells, a scratch assay was performed. As Figure 3A shows, MORC2 knockdown in both HuCCT1 and RBE cells was associated with decreased closure of the wound area compared with control cells. To explore whether MORC2 affects the invasive ability of CCA cells, transwell assays were conducted. The results showed that MORC2 knockdown dramatically attenuated the invasive capacities of HuCCT1 and RBE cells compared with control cells (Figure 3B). To further explore the underlying molecular mechanism of MORC2 in the epithelial–mesenchymal transition (EMT), western blot analysis was performed to examine the EMT associated protein markers in MORC2-downregulated HuCCT1 and RBE cells. The results demonstrated that MORC2 knockdown significantly induced the expression of the epithelial marker E-cadherin (Figure 3C). Conversely, the expression levels of the mesenchymal markers N-cadherin and vimentin were dramatically impaired in response to MORC2 knockdown (Figure 3C). Additionally, MORC2 knockdown significantly decreased the expression levels of Slug, the EMT-inducing transcription factor (Figure 3C).

### Knockdown of MORC2 suppresses tumor growth and liver metastasis *in vivo*

**Figure 4:**
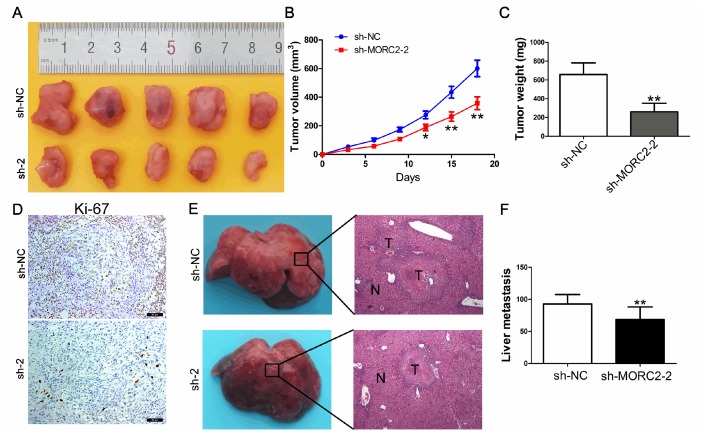
**Knockdown of MORC2 inhibits tumor growth and liver metastasis in vivo.** (**A**–**C**) Knockdown of MORC2 expression significantly inhibited CCA cell growth in nude mice, and the tumor weight (**B**) and tumor volume (**C**) were significantly reduced in the sh-MORC2 group compared to those in the sh-NC group. (**D**) Knockdown of MORC2 significantly reduced Ki-67 expression in vivo. (**E**) An experimental metastasis animal model was constructed by injecting MORC2 stable knockdown HuCCT1 cells into the distal tip of the spleen (Left). Representative images from each group are shown (Right). (**F**) The number of tumor nodules on the liver surfaces from the two groups is shown. **P < 0.01.

To further confirm the results of the experiments *in vitro*, a xenograft tumor growth assay was conducted. Cells (sh-NC and sh-MORC2-2) were subcutaneously inoculated into BALB/c nude mice. At 21 days after implantation, tumors harvested from MORC2 knockdown cells exhibited markedly decreased tumor growth rates, average volume and weight (Figure 4A–4C). IHC analysis demonstrated that MORC2 knockdown resulted in decreased expression levels of Ki-67 (Figure 4D). To evaluate the *in vivo* effect of MORC2 on liver metastasis, cells (sh-NC and sh-MORC2-2) were injected into the distal tip of the spleen. The results revealed that HuCCT1 sh-MORC2-2 cells exhibited dramatically decreased numbers of metastatic nodules in livers compared with those injected with HuCCT1 sh-NC cells (Figure 4E–4F).

### MORC2 is a target gene of miR-186-5p

**Figure 5:**
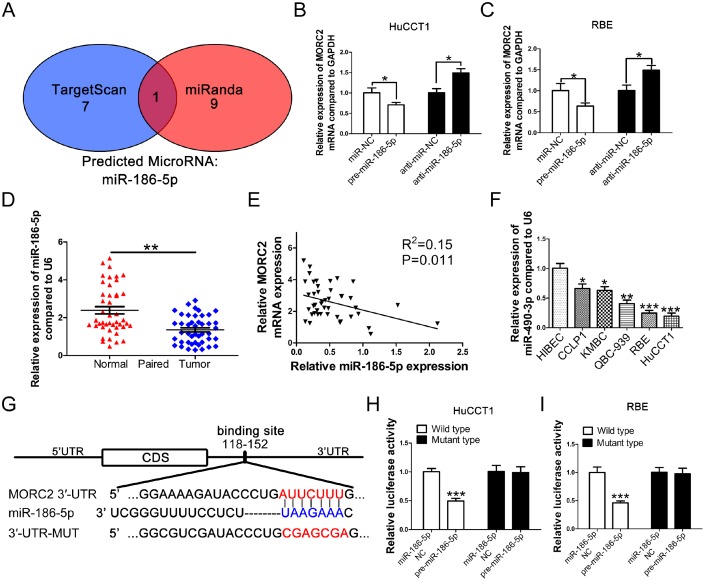
**MORC2 is a direct target of miR-186-5p in CCA cells.** (**A**) Analysis by TargetScan and miRanda target prediction algorithms revealed that the 3' UTR of MORC2 contains putative binding sites for multiple miRNAs. (**B**–**C**) qRT-PCR analysis of the expression levels of MORC2 after transfection with a miR-186-5p mimic or its inhibitor in HuCCT1 and RBE cell lines. (**D**) Relative expression levels of miR-186-5p in adjacent normal tissues and CCA tissues. (**E**) Correlation between miR-186-5p expression and MORC2 expression in clinical samples. (**F**) Expression levels of miR-186-5p in indicated CCA cell lines. (**G**) Schematic representation of potential binding sites of miR-186-5p with WT or Mut MORC2. (**H**–**I**) Luciferase activity of WT or Mut MORC2 after cotransfection of a luciferase construct fused with the wild-type or site mutant 3′-UTR of MORC2 and pre-miR-186-5p or miR-NC. All experiments were performed in triplicate, ***P < 0.001, **P < 0.01, *P < 0.05.

Whether MORC2 expression is modulated by microRNA at the post-transcriptional level in CCA remains unclear. In this study, we used two bioinformatic algorithms (miRanda and TargetScan) to identify potential miRNAs targeting MORC2. The data showed that miR-186-5p exhibited the greatest potential to modulate MORC2 mRNA expression (Figure 5A). To examine whether MORC2 expression was negatively regulated by miR-186-5p in CCA cells, HuCCT1 and RBE cells were transfected with miR-186-5p mimics and inhibitors. Then, MORC2 mRNA levels were detected. The results revealed that miR-186-5p overexpression markedly decreased MORC4 mRNA levels in both HuCCT1 and RBE cells (Figure 5B), while miR-186-5p downregulation led to increased MORC2 mRNA expression (Figure 5C). Next, we measured the miR-186-5p levels in 44 pairs of CCA specimens and adjacent nontumorous specimens. As Figure 5D shows, miR-186-5p expression was dramatically lower in CCA specimens. Furthermore, a negative correlation between miR-186-5p and MORC2 mRNA expression was found in CCA tissues (Figure 5E). Moreover, a lower miR-186-5p expression level was observed in CCA cell lines compared with that in HiBECs (Figure 5F). To further validate the direct binding relationship between miR-186-5p and MORC2, a luciferase assay was performed. Based on the bioinformatics analysis, one miR-186-5p binding site was observed to be located at 118-152 bp of the MORC2 3′-UTR (Figure 5G). The target sequence of the MORC2 3′-UTR (MORC2-wt-3′-UTR) and its mutant (MORC2-mt-3′-UTR) were cloned into a luciferase reporter vector. Cotransfection of miR-186-5p mimics and MORC2-wt-3′-UTR into HuCCT1 and RBE cells significantly impaired luciferase activity compared with the negative control cells. However, cells cotransfected with miR-186-5p mimics and MORC2-mt-3′-UTR exhibited no difference in luciferase activity (Figure 5H-5I).

### miR-186-5p is involved in MORC2-mediated CCA cell growth and metastasis

**Figure 6:**
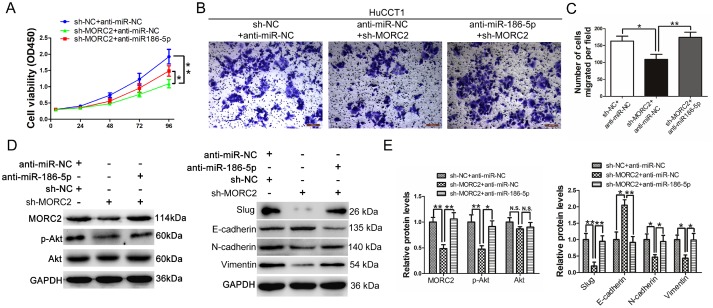
**miR-186-5p is involved in MORC2-mediated CCA cell growth and metastasis.** (**A**) CCK-8 analysis of cell viability after transfection with miR-186-5p inhibitor and sh-MORC2 plasmid in the HuCCT1 cell line. (**B**–**C**) Transwell analysis of cell invasive potential after transfection with miR-186-5p inhibitor and sh-MORC2 plasmid in the HuCCT1 cell line. (**D**–**E**) Western blot analysis of the levels of p-AKT, AKT, Slug, E-cadherin, N-cadherin and Vimentin after transfection with miR-186-5p inhibitor and sh-MORC2 plasmid in the HuCCT1 cell line. All experiments were performed in triplicate, **P < 0.01, *P < 0.05.

To explore whether miR-186-5p regulates CCA cell proliferation in a MORC2-dependent manner, we cotransfected HuCCT1 cells with miR-186-5p inhibitor and sh-MORC2 plasmid. The data revealed that the growth-inhibitory effect that resulted from MORC2 knockdown was partly restored by miR-186-5p silencing, as demonstrated by CCK-8 assays (Figure 6A). Similarly, HuCCT1 cells cotransfected with miR-186-5p inhibitor and sh-MORC2 plasmid demonstrated markedly higher capabilities to invade (Figure 6B–6C) compared to those transfected with sh-MORC2 plasmid alone. Moreover, cotransfection with the miR-186-5p inhibitor rescued the decreased MORC2 protein expression caused by the sh-MORC2 plasmid, and restored the reduced expression levels of p-Akt, Slug, N-cadherin and Vimentin (Figure 6D-6E). Additionally, the increased E-cadherin protein expression that resulted from shRNA silencing was reversed by cotransfection with the miR-186-5p inhibitor. Collectively, these data suggested that miR-186-5p regulated the function of MORC2, and could influence CCA cell proliferation and metastasis by regulating MORC2.

## DISCUSSION

CCA is one of the most malignant diseases worldwide. Uncontrolled tumor growth and distant metastasis remain huge obstacles to effective treatments. It is of vital importance to identify novel targeted molecules that contribute to the growth and metastasis of cancer cells. It is well documented that MORC2 inhibits the expression of several tumor suppressor genes, and thus promotes cancer progression [6]. Other studies demonstrated that MORC2 facilitates DNA repair and has a cytosolic role in lipogenesis and adipogenesis [4]. However, its functions in CCA are still unclear. In the present study, we reported that MORC2 was frequently upregulated in human CCA, and that high MORC2 expression was associated with poor prognosis of CCA patients. MORC2 exhibited tumor-promoting activities towards CCA cell growth and metastasis by activation of Akt signaling and EMT. Furthermore, the current study demonstrated that low miR-186-5p expression may partly contribute to MORC2 overexpression and facilitate the application of its oncogenic role in CCA.

It is well reported that EMT plays essential roles in invasiveness and metastasis in most types of cancers [14]. During EMT, cancer cells may lose cell-cell adhesion and obtain migratory capability to break away from neighboring cells to invade adjacent cell layers [15]. A recent report demonstrated that expression of the M276I mutant MORC2 in triple-negative breast cancer cells promoted Slug protein and mRNA expression, but not Snail, Twist, and Zeb1 [7]. In the present study, our data showed that MORC2 promoted CCA cell migration and invasion via activation of Slug, subsequently resulting in EMT evolution. It is interesting to note that the PI3K/AKT signaling pathway is involved in EMT in several types of cancers [16–17]. In our study, we found that knockdown of MORC2 in CCA cell resulted in dramatically decreased phosphorylation of Akt. Therefore, in the next step, the PI3K/AKT signaling pathway contributing to MORC2-induced EMT in the metastasis of CCA deserves further investigation.

Recently, dysregulated miRNAs have been shown to play either an oncogenic or tumor suppressor role in modulating multiple cancer progression [18]. Similarly, miR-186 is a cancer type-specific microRNA that has dual functions in tumorigenesis and cancer progression. For example, miR-186 has been reported to be overexpressed in endometrial, pancreatic, and cervical cancers [19–22], indicating an oncogenic role in these cancers. Conversely, miR-186 may function as a tumor suppressor in CRC and non-small cell lung cancer (NSCLC), because its overexpression impaired cell proliferation and metastasis of CLR and NSCLC cell lines *in vitro* [11, 23, 24]. In the current study, the data indicated that miR-186-5p expression was frequently downregulated in CCA specimens and cell lines, suggesting that miR-186-5p may function as a tumor-suppressor in human CCA. Functional experiments revealed that the targeting of MORC2 may be an important mechanism by which miR-186-5p exerts its anti-tumor function. It is worth noting that miR-186-5p can directly bind to the 3′-UTR of ZEB1 and reduce its expression, thus affecting the EMT process in CRC cells. In our study, rescue experiments also demonstrated that miR-186-5p affected the EMT process by regulating MORC2 expression. It is essential that future studies examine whether miR-186-5p regulates the EMT process by reducing ZEB1 expression.

In summary, our data revealed that MORC2 is upregulated in human CCA cell lines and specimens and that MORC2 knockdown can suppress CCA cell growth, migration, and invasion both *in vivo* and *in vitro*. Furthermore, high MORC2 expression is associated with poor 5-year survival. Regarding the mechanism, we found that MORC2 promotes CCA cell metastasis through the EMT process and enhances proliferation via the Akt signaling pathway, indicating its role as a novel therapeutic target in CCA treatment. Further studies are indicated to explore the function of MORC2 in CCA progression.

## MATERIALS AND METHODS

### Clinical samples and cell lines

A total of 44 pairs of CCA samples and their corresponding adjacent nontumorous tissue samples were obtained from the Sun Yat-sen University Cancer Center between February 2011 and November 2014. The inclusion criteria of all patients included in this study were: (a) definitive CCA diagnosis by histopathological evaluation; (b) curative resection, defined as tumor free at resection margins by histological examination; and (c) no adjuvant therapy was conducted before surgery. Clinical specimens were frozen in liquid nitrogen. All patients provided written informed consent. The protocols used in the study were approved by the institutional ethics review board of Sun Yat-sen University Cancer Center. The human CCA cell lines QBC-939 and RBE were purchased from the Type Culture Collection of the Chinese Academy of Sciences (Shanghai, China). Other CCA cells (KMBC, HuCCT1 and CCLP1) and the human intrahepatic biliary epithelial cell line (HIBEC) were preserved in our laboratory. RBE and CCLP1 cells were cultured in DMEM; HIBEC, HuCCT1, KMBC and QBC-939 cells were cultured in RPMI 1640 with 10% fetal bovine serum (FBS) (Invitrogen, Shanghai, China).

### RNA isolation and qRT-PCR assays

Total RNA from fresh specimens and cells was extracted using TRIzol reagent (Invitrogen, Shanghai, China). The PrimeScript RT reagent kit (Takara, Dalian, China) was then used for the reverse transcription of 1 μg of RNA according to the manufacturer’s instructions. qRT-PCR assays were conducted using SYBR Premix Ex Taq (Takara) according to the manufacturer’s instructions. The primers for MORC2 were: 5′-CACACAAATTCAACC ACTCACG-3′ (sense) and 5′-GGTCCTCTCGTCTTTCTGCA T-3′ (antisense); The primers for GAPDH were: 5′-GGGAGCCAAAAGGGTCAT-3′ (sense) and 5′-GAGTCCTTCCACG ATACCAA (antisense). MiRNAs were reverse transcribed using the miRcute Plus miRNA First-Strand cDNA Synthesis Kit (TIANGEN, Beijing), and the miRcute Plus miRNA qPCR Detection Kit (TIANGEN, Beijing) was used to detect the expression of miR-186-5p. The primers for miR-186-5p were: 5′-GCGGATCCGAGCCATGCTTATGCTACTG-3′ (sense) and 5′-GCGCGGCCGCCCAGGTATATGGCA-3′(antisense). The 2 ^-ΔΔCT^ method was used to calculate the relative expression levels of target genes and miRNAs.

### Western blot analysis and antibodies

Clinical specimens and total cell lysates were isolated using RIPA lysis buffer (Beyotime, Jiangsu, China) supplemented with the protease inhibitor phenylmethanesulfonyl fluoride (Beyotime, Jiangsu, China). Equal amounts (40 μg) of proteins were separated by 10% polyacrylamide gel electrophoresis, and then transferred to polyvinylidene fluoride membranes (Millipore, Billerica, MA, USA). After blocking in 5% nonfat milk for 2 h, the membranes were incubated overnight at 4 °C with the primary antibodies. Following incubation with a secondary antibody at room temperature, the proteins were visualized using an enhanced chemiluminescence detection kit reagent (Millipore, Billerica, USA). The following primary antibodies were applied for western blot analysis in this study: anti-MORC2 (1:500, ab203062), anti-GAPDH (1:10000, ab181602), anti-E-cadherin (1:10000, ab40772), anti-N-cadherin (1:1000, ab76057) and anti-Vimentin (1:2000, ab92547) (Abcam, Cambridge, MA, USA); anti-Slug (1:1000, #9585), anti-Akt(1:1000, #4685) and anti-p-Akt(1:1000, #4060) (CST, Danvers, MA, USA).

### Immunohistochemistry assays

IHC in this study was performed as previously described [12]. The MORC2 (ab203062, Abcam, Cambridge, MA, USA) and Ki-67(ab156956, Abcam, Cambridge, MA, USA) primary antibodies were used at a 1:150 dilution in the IHC experiment. The immunoreactivity score was calculated as previously reported [25].

### Lentiviral vector production and construction of stable cell lines

Lentiviral vectors encoding MORC2-target shRNA and negative control shRNA were purchased from GeneChem (Shanghai, China). Target sequences specific for MORC2 genes were as follows: sh-MORC-1 (5'-GCGGAACAUUGGUGAUCAU-3′), sh-MORC-2 (5'-GGAGCCUACACACAACAAA-3′) and sh-MORC-3 (5'-GCAGCUGAGUGCUAUGAA U-3′). The shRNA sequence used as the negative control (sh-NC) was 5'-UUCUCCGAA CGUGUCACGU-3′. HuCCT1 and RBE cells were transfected with lentiviral vectors according to the manufacturer’s instructions. Stable cell lines were selected using 1 μg/ml puromycin.

### Cell proliferation assays

The ability of CCA cell proliferation was explored using CCK-8 (Dojindo Laboratories, Kumamoto, Japan) and EdU assay (Life Technologies) kits according to the manufacturer’s instructions. For colony formation assays, 1000 cells/well were seeded in 6-well plates in triplicate and cultured at 37°C and 5% CO2 in a humidifed chamber for 2 weeks. Colonies were stained with 1% crystal violet and counted.

### Cell migration and invasion assays

To assess cell migration ability, CCA cells were seeded in 6-well plates, and 200 μl pipette tips were used to make a scratch. The wounded monolayer was washed and reincubated with serum-free media. The data were measured at 0 h and 36 h. For invasion assays, the upper chambers (Corning, NY, USA) were coated with Matrigel (BD Biosciences, San Jose, CA, USA) for 3 h. Then, 1×10^5^ CCA cells were resuspended in 200 μl of serum-free media, and seeded on the upper champers. After 24 h, cells in the upper chambers were removed and cells in the lower membranes were fixed using 4% PFA and stained with 1% crystal violet. Experiments were conducted in triplicate.

### Luciferase reporter assays

HuCCT1 and RBE cells were seeded in 24-well plates, and cotransfected with the indicated luciferase reporter plasmid (200 ng), miR-186-5p mimics/miR-NC (100 nM) and Renilla-encoding plasmids. After a 24 h transfection, the cells were collected and lysed. The firefly and renilla activities were analyzed by the Dual-Luciferase Reporter Assay System (Promega, Madison, WI, USA). Each transfection was performed in triplicate.

### *In vivo* proliferation and metastasis experiments

A total of 10 athymic female nude mice (6 weeks old) were purchased from Vital River Lab Animal Technology Co., Ltd. All animal experiments were performed in accordance with the guidelines of the National Institutes of Health. To explore the tumorigenic effects of MORC2 *in vivo*, HuCCT1 cells were subcutaneously injected into the right side of the nude mice (5×10^6^ cells/mouse, 5 nude mice per group). Tumor size was measured every 4 days. The mice were sacrificed after 3 weeks and the tumors were harvested for analysis. To determine the role of MORC2 in tumor metastasis *in vivo*, HuCCT1 cells were injected into the distal tip of the spleen after laparotomy (1×10^6^ cells/mouse, 4 nude mice per group). All mice were sacrificed after 5 weeks. The livers were dissected t for further analysis.

### Statistical analysis

The results are presented as the mean ± SD from at least three independent experiments, and were analyzed with SPSS 22 (IBM Corp, Armonk, NY) or GraphPad Prism 5 (GraphPad Software, San Diego, CA, USA). The two-tailed Student's t-test was conducted to analyze the differences between two groups. Pearson’s correlation coefficient was used to examine the association between MORC2 and miR-186-5p expression in clinical specimens. χ2 tests were used to compare the frequencies of categorical variables. Survival data were determined by the Kaplan-Meier method and difference among each group were calculated by the log-rank test. P < 0.05 was considered statistically significant.
